# Depletion of coastal predatory fish sub-stocks coincided with the largest sea urchin grazing event observed in the NE Atlantic

**DOI:** 10.1007/s13280-020-01362-4

**Published:** 2020-07-27

**Authors:** Kjell Magnus Norderhaug, Kjell Nedreaas, Mats Huserbråten, Even Moland

**Affiliations:** 1 Institute of Marine Research IMR, Norway, Nye Flødevigveien 20, 4817 His, Norway; 2 University of Oslo Norway, Oslo, Norway; 3 Institute of Marine Research, Norway, Nordnesgaten 33, 5005 Bergen, Norway; 4 Institute of Marine Research, Norway, Nordnesgaten 50, 5005 Bergen, Norway; 5 Institute of Marine Research, Norway, Nye Flødevigveien 20, 4817 His, Norway; 6 Centre for Coastal Research (CCR), University of Agder, Kristiansand, Norway

**Keywords:** Coastal fisheries, Fisheries management, Kelp forest, Regime shifts, Sea urchin grazing

## Abstract

In this contribution, we propose fishery driven predator release as the cause for the largest grazing event ever observed in the NE Atlantic. Based on the evolving appreciation of limits to population connectivity, published and previously unpublished data, we discuss whether overfishing caused a grazer bloom of the sea urchin (*Strongylocentrotus droebachiensis*) resulting in overgrazing of more than 2000 km^2^ kelp (*Laminaria hyperborea*) forest along Norwegian and Russian coasts during the 1970 s. We show that coastal fisheries likely depleted predatory coastal fish stocks through modernization of fishing methods and fleet. These fish were important predators on urchins and the reduction coincided with the urchin bloom. From this circumstantial evidence, we hypothesize that coastal predatory fish were important in regulating sea urchins, and that a local population dynamics perspective is necessary in management of coastal ecosystems.

## Introduction

Hunting and fishing by humans have decimated large predator populations in many coastal areas globally (Jackson et al. 2001). Removal of predators high in the food web have caused ecosystem collapse with cascading effects through several food web levels. In the absence of predators, grazers may flourish, which in turn leads to overgrazing of the primary producers. Benthic primary producers like kelp and seagrasses are engineering species that provide ecosystem services, including habitats, that promote ecological stability (Teagle et al. 2017). When removed, desert-like barren grounds can prevail for decades (Ling et al. 2015). Recent development of molecular tools and increasingly sophisticated use in population genetics have brought new insight into reproductive patterns and connectivity between marine populations. Only during the last decade these tools have changed our understanding on population dynamics of important fish stocks including coastal Atlantic cod (*Gadus morhua*) (Jorde et al. 2007; Knutsen et al. 2011; Dahle et al. 2018). This understanding throw light on why management of coastal fish stocks in many cases has failed in preventing ecosystem collapse.

Historically, a view prevailed of coastal stocks being supported and replenished by near inexhaustible offshore resources, with a constant surplus of larvae, and starvation during vulnerable stages being the most important impediment to stable recruitment (Hjort 1914). However, more complex models have been developed to understand recruitment variability and fluctuations in fish populations (see Houde 2008). Recently, molecular tools have facilitated the discovery that along Norway’s convoluted coastline, limited gene-flow may result in local populations (e.g., Jorde et al. 2007; Knutsen et al. 2011; Quintela et al. 2020). In coastal Atlantic cod, such patterns in population structure are driven by processes such as adult movement and spawning behavior, placement of propagules by spawners, retention of propagules by local hydrographical phenomena and local retention of fish larvae (Ciannelli et al. 2010; Skjaeraasen et al. 2011; Rogers et al. 2014; Huserbråten et al. 2018; Barth et al. 2019). Similar findings are presently underway for a suite of species, with coastal and fjordic affinities and widely different life histories. This shift in our appreciation of implicit vulnerabilities challenges traditional stock assessment models and classical management—assuming discrete populations within large administrational areas—calls for sub-stock-specific management recommendations (see e.g., Reiss et al. 2009; Lindegren et al. 2013; Dahle et al. 2018; Svedäng et al. 2018).

Importantly, an appreciation of local-scale processes in maintenance of coastal fish populations has implications for assessing the functional role of predatory fish and the impact of fisheries on coastal ecosystems (Hammerschlag et al. 2019). This needs to be taken into account in sustainable fisheries management as past management actions have failed to adapt to prevailing biological processes (Francis et al. 2007; Svedäng et al. 2010; Cardinale et al. 2017). Although both functional diversity and redundancy is thought to be high for demersal fish in the Barents Sea region (Aune et al. 2018), limited research has been directed towards ecosystem functioning and possible harvest induced alterations in the Arctic.

Aquatic ecosystems characterized by site-attached species with limited connectivity and strong biological interactions are likely to harbor less functional redundancy than more ‘open’ ecosystems (Teichert et al. 2017). Whether separated by physical, genomic or behavioral barriers (see Barth et al. 2019), subpopulations are likely more vulnerable to local depletion than large widespread fish stocks (André et al. 2016; Cardinale et al. 2017; Gunnarson et al. 2019). Consequently, coastal fisheries targeting local sub-stocks of predatory fish may have resulted in far greater ecosystem effects than anticipated historically. This recognition may throw light on one historic event that took place five decades ago and with major repercussions for coastal production and diversity. A large-scale bloom of sea urchins *Strongylocentrotus droebachiensis* along Norwegian and Russian coasts took place and some 2000 km^2^ kelp *Laminaria hyperborea* forests were grazed down and turned into a marine desert, or so-called barren ground (Sivertsen 1997). To date, the causes of this bloom of grazers remain largely unknown.

Here, we present previously unpublished data and discuss the hypothesis that past coastal fisheries removed predatory fish controlling sea urchin populations and thus triggered an ecosystem collapse. No systematic collection of fisheries statistics for coastal stocks exist from the period before and during the grazing event. However, we have digitalized historic and previously unpublished fishery sales notes statistics (data provided by SSB, Statistics Norway) and combined this with anecdotal knowledge to increase the spatiotemporal resolution of these data. Together with target species’ diet data, these datasets provide the first opportunity to evaluate this hypothesis.

## Methods

To identify potentially important green sea urchin predators among fishery target species, our main source of information is the extensive data compilation by Planque et al. (2014) to determine food web links in the Barents Sea. Their data preparation protocol categorize predator–prey relationships from 1 to 4. From this we identified four candidate fishery target species (Atlantic wolffish, spotted wolffish, Atlantic cod and haddock, see Table 1) that were listed as ‘category 1′ predators of green sea urchin. For three of these species (Atlantic wolffish, haddock and Atlantic cod) we obtained, digitalized and plotted landings data for the relevant coastal areas and time period, from 1950 to present (fishery sales notes statistics provided by SSB, Statistics Norway). Accounts of Norwegian fisheries and landings can be found dating back to 1866. Until 1977 these data were reported by coastal district and county where the catches were landed and are only available on paper. Since 1977, Norwegian landings statistics are available in electronic format, by county and statistical area, as well as inside/outside 12 nautical miles (nm) from the baseline. Since 2000, landings statistics have a much higher resolution, i.e., down to fishing vessel, port and company level.

For Atlantic wolffish and haddock, the landing statistics per county are used until 1980, and per statistical area inside 12 nm during 1980–2018. Hence, there may be some catches from outside 12 nm included in the county-based 1950–1979 landings statistics, and direct comparison between before and after 1980 should be made with caution. To ‘ground-truth’ our interpretation of landings patterns for Atlantic wolffish, we conducted informal interviews with three fishers with first-hand experience from the fishery, in space and time. Two (R. Rånes, I.J. Husby) were selected from IMRs reference fleet (administered by author KN), and the third (A.J. Trondal) was recruited based on IMR local knowledge in Finnmark.

Based on otolith shape, the proportion of coastal cod can be distinguished from northeast arctic cod (NEAC) (Rollefsen 1933). Otolith samples have been collected from cod landings since 1984 and were therefore available from 1984 to 2018. During 1914–1983, Norwegian landings statistics of cod are categorized differently. The category “bank cod” and “fjord cod” (or “other cod”) is presented as a proxy for coastal cod landings during 1950–1961. Between 1970 and 1983 landing statistics of cod are mainly categorized as “cod”, without distinguishing landings spatially, temporally or biologically and are therefore not presented. For all three fish species, Welch Two Sample t-tests were performed to compare landings before and after the sea urchin outbreak.

### Coastal fisheries and ecosystem collapse globally

Blooms of grazers escaping predator control have been reported from other coastal areas (Estes et al. 1998; Jackson et al. 2001; Steneck et al. 2004) and have increased in frequency, mainly due to overfishing and climate change (Ling et al. 2015). Among the most well-known consequences are the hunting of sea otters in Alaska (Estes et al. 1998) and fisheries in the Gulf of Maine (Steneck et al. 2004) both leading to blooms of grazers and denuding of kelp forests. Industrial fisheries started in the western world from the 1930s, and by the 1960s had developed enough capacity to overfish stocks—resulting in cascading perturbations in many coastal food webs globally (Jackson et al. 2001). In Maine, developing fishing technology and onboard refrigeration improved the efficiency of the fleet to target spawning coastal cod and other predatory fish. By the 1960s, overfishing of large predatory fish including Atlantic wolffish, coastal Atlantic cod and haddock caused blooms of *S. droebachiensis* along the coast of Maine after more than 4 000 years of predatory fish domination (Steneck et al. 2004). The resulting overgrazing of kelp forests and formation of barren grounds lasted until fisheries decimated urchin populations in the mid-1990s (Steneck et al. 2013). As a consequence, kelp forests recovered. Predatory fish were still scarce, and a new apex predator expanded in the vacant niche, the crab *Cancer borealis*.

### The only large-scale grazing event in the NE Atlantic

Blooms of green sea urchins *S. droebachiensis* were first observed by fishermen in the early 1970s along the coast of Mid- and North Norway. A 2 000 km^2^ large area formerly dominated by kelp *L. hyperborea* forests from Smøla (63° N) in the south to Finnmark (71° N) and into Russia in the north were denuded and turned into sea urchin dominated barren grounds (Sivertsen 1997). This constitutes the first known large-scale grazing event in the NE Atlantic from any available source going back almost 150 years (Norderhaug and Christie 2009). While kelp forests have recovered in the southern part of the barren grounds, sea urchins still dominate most of the denuded areas five decades later (Christie et al. 2018).

Removal of kelp forests have severe implications for coastal production and diversity, provision of habitats and nursery areas for coastal fish (Filbee-Dexter and Scheibling 2014) and sequestration of carbon in the affected areas (Krause-Jensen and Duarte 2016). Atlantic wolfish, haddock, coastal cod and other coastal fish associated to kelp forests have remained at low levels in the grazed areas and are expected to impact seabirds (Christensen-Dalsgaard et al. 2017) and marine mammals (Bjørge 1995).

In the period after the urchin bloom, several hypotheses were put forward, including regulation of sea urchin populations by the endoparasite *Echinomermella matsi* (Hagen 1987), amoebic disease (Christie et al. 1995), and temperature (Sivertsen 2006). These hypotheses were later rejected or questioned: Screenings revealed no evidence for microbial infections explaining mass mortality of sea urchins observed at 66°44′ N (Christie et al. 1995), and *E. matsi* could not account for local population crash episodes of *S. droebachiensis* (several local studies including, Stien et al. 1995). While *S. droebachiensis* is a cold-water species and prolific during cold periods, temperature alone cannot explain the outbreak (Anon. 2002; Norderhaug and Christie 2009). Thus, the drivers responsible for the “regime shift” have never been properly understood.

### Diets of predatory fish

The importance of predators in regulating prey populations depend on biological interaction strength. The strong controlling impact *S. droebachiensis* has on kelp *L. hyperborea* is widely documented including Norwegian waters (Ling et al. 2015). In Maine and other coastal areas, the strong impact from predatory fish like coastal cod, haddock and Atlantic wolffish in regulating *S. droebachiensis* is well documented (Steneck et al. 2013). Also, in north Norwegian coastal waters available literature suggest these predatory fish are important predators on *S. droebachiensis* (Planque et al. 2014; Strand 2019). Benthic predatory fish are typically generalists and their prey will depend on what is available. Diets will therefore vary in time and space (Jiang and Jørgensen 1996). We do not know the diet of our focal fish species (Table 1) in the period prior to the grazing event, but *S. droebachiensis* may well have been a preferred food item because of its high nutritional value, especially when gonads are well developed (Tam et al. 2016). Today, the green sea urchin *S. droebachiensis* is considered the main prey of Atlantic wolffish (*Anarhichas lupus*) in the grazed region (Falk-Petersen et al. 2010), and also important prey for the spotted wolffish (*Anarhichas minor,* Simpson et al. 2013). Both wolffish species are vulnerable to harvest due to late sexual maturity (6–7 and 7–10 years for *A. lupus* and *A. minor*, respectively), site fidelity and life history attributes that confer vulnerability to overharvesting (see Table 1). Coastal Atlantic cod *Gadus morhua* is a typical opportunistic generalist predator with variable diet including fish and invertebrates, and *S. droebachiensis* is important prey in northern Norway fjord areas where they are abundant (Enoksen and Reiss 2018).

**Table 1:** Fishery target species categorized as principal predators on green sea urchins *S. droebachiensis* (‘category 1′ according to Planque et al. 2014)

Species	Indices of vulnerability	Gear	Modernization	References
Antlantic wolffish *Anarhicas lupus*	Internal fertilization, late maturing, low fecundity, paternal care of demersal eggs, homing to feeding and spawning grounds	Longline Bottom trawl^b^ Gillnet	ABCD	Eliassen et al. (1981), Keats et al. (1985), Falk-Petersen et al. (2010), Simpson et al. (2013) and Gunnarson et al. (2019)
Spotted wolffish *Anarhicas minor*	Internal fertilization, late maturing, low fecundity, paternal care of demersal eggs	Longline Bottom trawl^b^ Gillnet	ABCD	Eliassen et al. (1981), Gunnarson et al. (2008) and Simpson et al. (2013)
Norwegian coastal cod *Gadus morhua*	Spawning aggregation, spawning site fidelity, population structure	Gillnet Longline Handline Bottom trawl Danish seine	ABCE	Jorde et al. (2007), Skjaeraasen et al. (2011), Dahle et al. (2018) and Enoksen and Reiss (2018)
NEA haddock *Melanogrammus aeglefinus*	Spawning aggregation, Population structure^a^	Gillnet Longline Handline Bottom trawl Danish seine	ABCE	Jiang and Jørgensen (1996), Reiss et al. (2009), González-Irusta and Wright (2016) and Tam et al. (2016)

Indices of vulnerability: biological, life cycle or life history attributes with consequences for the species’ vulnerability to harvesting. Gear: mode of capture/fishing gear directly or indirectly affecting the target species. Modernization: technological development and demand (1960s–80 s) affecting targeting of the species (see *Notes* at bottom of table)

A = increased engine power; B = increased vessel size; C = introduction of nylon fiber; D = advent of market/demand, E = introduction of the hydraulic net hauler/line hauler

^a^Spatial scale of population structure poorly known (Reiss et al. 2009)

^b^Wolffish are by-caught in bottom trawling, and bottom trawling is detrimental to wolffish habitat

**Fig. 1:**
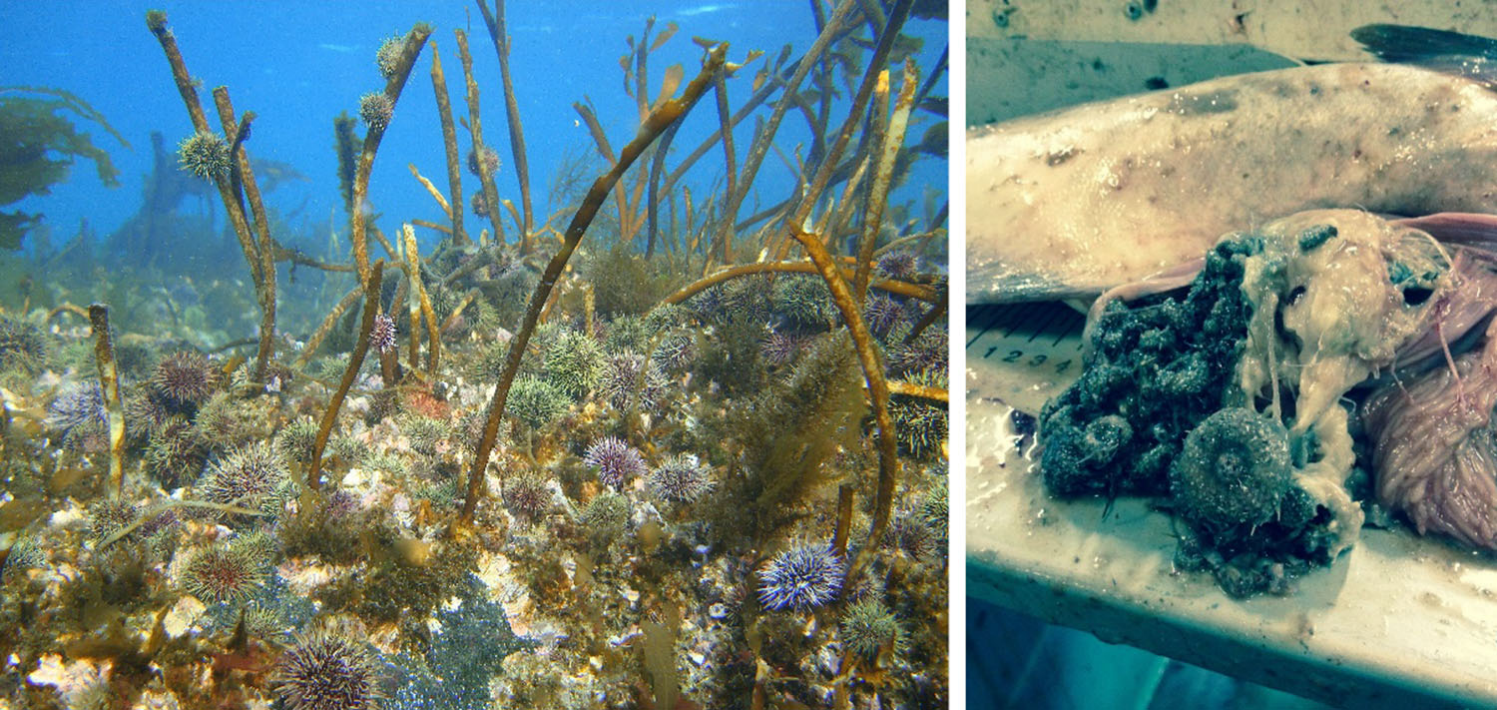
Green sea urchins *Strongylocentrotus droebachiensis* grazing kelp *Laminaria hyperborea* at Hammerfest (left side, Norway 71° N, photo: Stein Fredriksen) and green sea urchins in a haddock stomach sample (Photo: Hans Kristian Strand)

Haddock *Melanogrammus aglefinus* has a variable diet dominated by crustaceans and small echinoderms (Jiang and Jørgensen 1996; Tam et al. 2016) including *S. droebachiensis*. Being a choosier benthic feeder than Atlantic cod, some haddock individuals might specialize as urchin foragers and utilize periods with high abundance of post-settlement stage urchins (see Fig. 1). Together, Atlantic cod and haddock—would likely have exerted significant mortality on urchins throughout the affected area when abundant.

### Development of Norway’s coastal fisheries

Prior to the 1930s Norway’s fisheries were still dominated by handline and other gear that could be hand-hauled by fishers from traditional- and smaller-motorized fishing vessels. By the onset of World War II, the fleet had entered a period of rapid technical development. Modern seine was introduced in the herring-, sprat- and saithe fisheries, and the first Norwegian trawlers had been operating since 1936. To bolster the coastal fisheries, Norwegian government offered loans and subsidies for boat building and repairs, which led to a sharp increase in the capacity (Gerhardsen 1945). Despite increased capacity, less fish was brought to market during the war as subsistence fishing dominated. Landings dropped from one million metric tons in 1940 to ≈ 600 thousand tons in 1945. With peace in 1945, the coastal fishery expanded rapidly with a surge in boat building and participation. The number of registered vessels increased from ≈ 30 thousand in 1945 to an all-time high of 41 433 vessels in 1960 (Steinset 2018). Introduction of nylon in the late 1950s, and the hydraulic power block in the 1960s gave single fishing vessels the ability to haul deeper and longer line-sets and gill-nets, as well as seines that previously required large teams (Benum 2015). The technological revolution resulted in an unprecedented and unchecked increase in fishing effort, that eventually drove the mighty spring spawning herring (*Clupea harengus*) to collapse in the late 1960s (Dragesund et al. 2008).

**Fig. 2:**
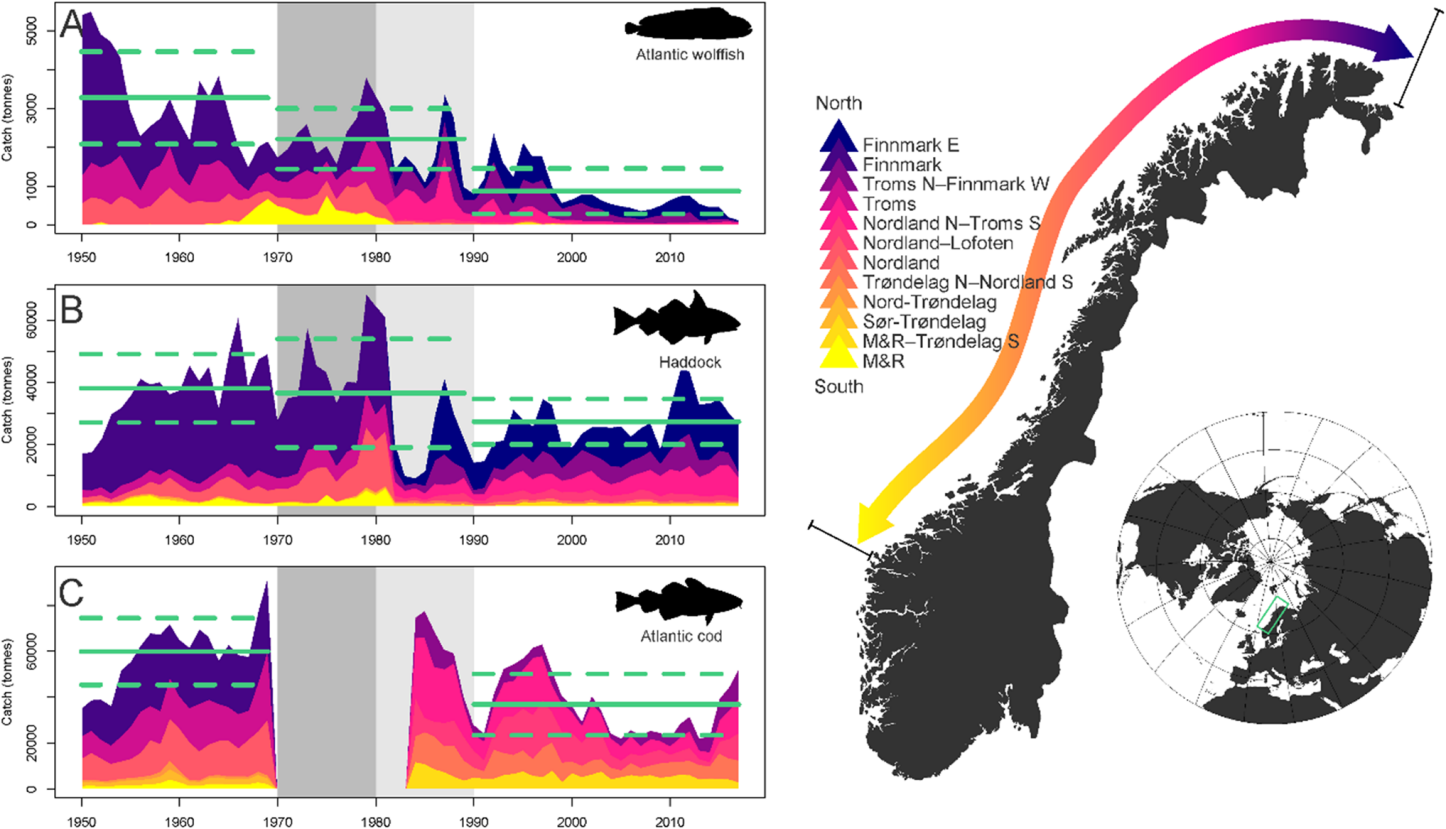
Spatiotemporal development of coastal fisheries for a Atlantic wolffish (*A. lupus*), b haddock (*M. eaglefinus*) and c Atlantic coastal cod (*G. morhua*) shown as regional landings (darker color with increasing latitude) available for the period 1950–2018 (see Methods) within the area where sea urchins (*S. droebachiensis*) bloomed (Norderhaug and Christie 2009). The green lines show average landings (± SD) before, during urchin overgrazing and after sea urchins started to retreat. During 1970–1980 (dark gray period) urchin populations bloomed and barren ground area coverage peaked. During 1980–1990 (light gray period) sea urchins gradually retracted while kelp recovered in the southernmost part of the barren area. Only total landings data of cod including Barents Sea cod are available during 1970–1983

Fisheries landing statistics digitalized and presented herein (described in the Method section) indicate depletion of predatory fish stocks in the decades before grazers bloomed and the coastal ecosystem collapsed. Landings of Atlantic wolffish (± SD) decreased significantly from 3268 (± 1187) tons before the grazer bloom (1950–1969) to 2215 (± 777) tons during the bloom (1970–1989) and to 869 (± 584) tons after sea urchins started retreating (1990–2017, Fig. 2, *t *= 8.35, df = 25.6, *p *< 0.0001 when comparing before and after). For haddock, annual landings were significantly reduced from 38 128 (± 11 014) tons before, to 36 561 (± 17 443) during and 27 376 (± 7324) tons after the bloom (*t *= 3.81, df = 30.7, *p *= 0.0006). The data, further, indicate increasing fisheries in the north as catches were reduced in the south. For Atlantic cod, coastal catches were significantly reduced from 59 932 (± 14 520) tons before (1950–1969) to 36 856 (± 13 279) tons after (1984–2017, *t *= 5.62, df = 38.8, *p *< 0.0001). For wolffish– primary predators on urchins—interviews with fishers conducted by the authors corroborate the pattern indicated by landings data. While demand and market for cod and haddock was well established for centuries, neither Atlantic nor spotted wolffish were targeted until the 1950s as they were considered unpalatable and unmarketable by Norwegian fishers and consumers. Throughout the 1950s and 1960s this changed as demand for these species drove prices up and opened a lucrative fishery that fishers could exploit during spring and summer, the off-season for the more established fisheries. Long-lines baited with squid were set in shallow water where wolffish were available—sometimes yielding catches where “every hook held a wolffish” when gear was deployed in virgin grounds (R. Rånes, pers. comm., see Methods). Atlantic wolffish landings peaked in the mid-1950s with well over 5000 metric tons annually. In the period 1962–1964, more than 3000 tons was still being landed annually in the affected areas, increasingly from the northernmost areas. By the late 1960s, Atlantic wolffish were gone from fjords and lagoons in the Bodø area (Nordland—Fig. 2, landings in orange), with no sign of recovery to date (R. Rånes, pers. comm.). Throughout the 1970s, wolffish species became high-end seafood also for Norwegian consumers (Eliassen et al. 1981). Over the period, the landings data indicated that stocks of coastal *A. lupus* underwent serial depletion from south to north. By the late 1980s, all but the northernmost areas were fished out. According to the complimentary interviews, the Nordland N—Troms S coastal section (Fig. 2, landings in light purple) yielded good catches from outer coastal areas on virgin grounds (< 100 m depth) during the 1980s to 90 s (I.J. Husby, pers. comm.). From Finnmark (Fig. 2, landings in purple), shallow coastal areas yielded good catches in a valuable spring fishery during the years 1997–1999 (A.J. Trondal, pers. comm., see also, Strand 2019). The conspicuous reduction in catches of all three predatory fish stocks during the 1980s was not related to regulation—as no or few regulations were in place until 1989—and therefore most likely resulting from stocks having been depleted throughout the historical fishing grounds. Other possible ecological reasons for the observed decline in landings include alterations in habitat quality or food availability. Bottom trawling would be the most likely driver of habitat alterations, especially detrimental to wolffish habitat (Table 1). Assessing the total impact of bottom trawling—also including international fishing vessels operating as near as 4 nmi from the Norwegian coast until the 1970s (A.H. Hoel, pers. comm.)—was beyond the scope of this study. It is possible that the fisheries-induced collapse of spring spawning herring in the late 1960s (see Dragesund et al. 2008), did confer changes in food availability to coastal predatory fish stocks in succeeding years. The consequences of the putative temporary loss of this trophic link would be worth exploring in future research. Lastly, the observed decline in landings could be affected by alteration in fisher behavior. Towards the end of the 1970s and for succeeding years, the Joint Russian-Norwegian Fisheries Commission agreed to introduce annual cod quotas, albeit insufficient to stop a positive trend in fishing mortality until the late 1980s (see Yaragina et al. 2011 and references therein). However, to the best knowledge of the authors, the degree to which this agreement brought about a shift in fisher behavior is unclear and does not preclude our conclusions.

## Discussion

**Fig. 3:**
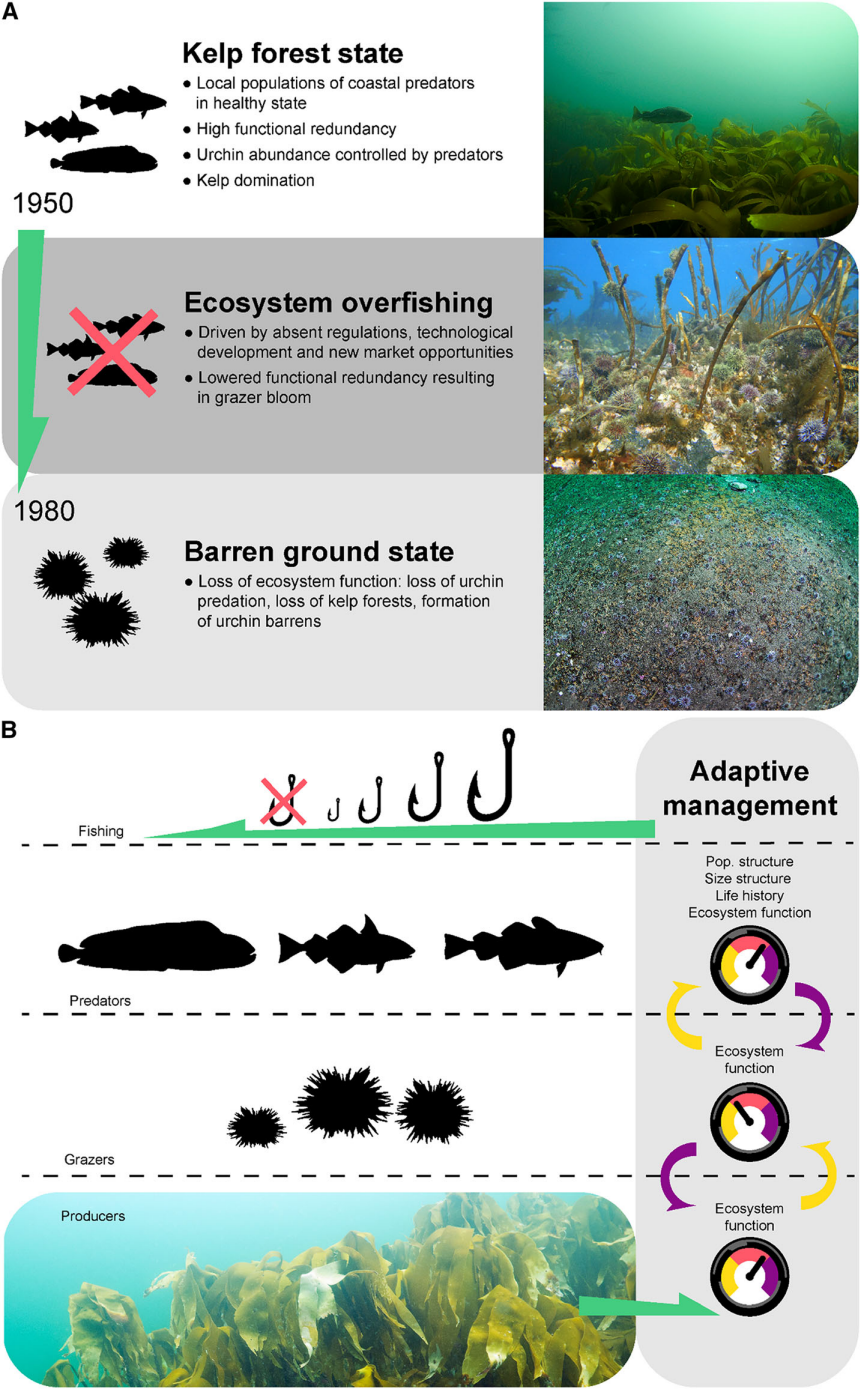
Hypothesized drivers of the regime shift and idealized ecosystem-based adaptive management. **a** Prior to 1950, the fishing pressure did not deplete coastal demersal predators of sea urchins to levels that threatened functional redundancy. From 1950, an unregulated coastal fishery, rapid technological development of the fleet and increased price of wolffish led to overfishing, serial depletion and loss of ecosystem function (ecosystem overfishing, Murawski 2000). By 1980, the barren ground state reached its maximum spatial coverage (photos from the top: J Thormar, S Fredriksen, E Svensen). **b** Adaptive ecosystem-based management takes species- and sub-stock-specific vulnerabilities into account. Environmental monitoring (symbolized by gauges) on species, sub-stock, and ecosystem state and function is used to revise and tune management and protection tools (symbolized by fish-hook size). Conservation of sub-stock biomass and size-structure prevent urchin blooms and support healthy ecosystems dominated by kelp forests (photo: J Thormar)

Herein we have summarized and evaluated available evidence on the potential historical role of local fisheries in depleting predatory fish stocks along coastal Norway (Fig. 3a). We have shown that these predatory fish are important predators on the green sea urchin *S. droebachiensis,* the principal grazer in the coastal ecosystem. We have also shown that the reduction of fish stocks coincided with the largest historical bloom of *S. droebachiensis* observed in coastal Norway for almost 150 years (Norderhaug and Christie 2009), resulting in large-scale denuding of kelp *L. hyperborea* forests into barren grounds during the 1970s.

Based upon this—and the evolving understanding of limits to connectivity in coastal populations, we hypothesize that coastal predatory fish are important in regulating sea urchin populations and that overfishing has caused ecosystem collapse by releasing the principal grazer from predatory control. The resulting large-scale overgrazing of *L. hyperborea* kelp forest had dramatic consequences for the coastal ecosystem structure and hence also on its diversity, production, functional redundancy and other ecosystem services, with effects far exceeding the loss of kelp forests itself (Norderhaug and Christie 2009).

While the evidence we present is circumstantial, the course of events is similar to other coastal areas and Norwegian ecosystems that seem to have collapsed in the same way (Estes et al. 1998; Jackson et al. 2001; Steneck et al. 2004). The realization of coastal fish stocks’ vulnerability to fishing may aid in understanding the failures of the past with implications for what can be defined as sound and sustainable management (see ‘Indices of vulnerability’ in Table 1). Small, semi-isolated populations, and particularly slow-growing and long-lived species, need strong regulation since the risk of local extinction for populations by random disturbances increases with isolation but decreases with population size and reproductive potential (see e.g., Begon et al. 2005). The combination of fish traits and the convoluted Norwegian coastal topography therefore calls for caution when it comes to fishing pressure (see Dahle et al. 2018).

Coastal fisheries are integral in Norwegian culture and rural development policy. The layman’s view is that such fisheries—based on smaller vessels—is a sustainable part of a ‘natural’ utilization of coastal production by small communities, which do not pose a threat to fish stocks in the same way as large-scale fisheries by industrial trawlers. This was probably an important reason why the management historically focused on assessment and advice for offshore stocks. Until 1989, the management of coastal stocks mainly emphasized regulating vessel size and gear standards, rather than monitoring stock sizes and vulnerabilities (Hylen et al. 2008; Gullestad et al. 2014). This phenomenon in fisheries management, termed the ‘easy restriction syndrome’ by Cardinale et al. (2017), refers to using the least controversial restriction without basis in data. The failure of taking the modernization of this previously unmanaged fishing fleet into consideration may have been catastrophic for several small and/or local fish stocks. To this day, the predatory fish stocks has failed to recover (Fig. 2), and similar to the Gulf of Maine coast and facilitated by ocean warming, predatory crab populations expand and seem to have taken over the niche as apex predators (Fagerli et al. 2014; Christie et al. 2018).

In our view, there is an urgent need for bridging the gap between research and policy. Successful management of coastal fish stocks hinges on good information on life history, reproductive strategy, population structure (connectivity and source sink dynamics), and functional role in the ecosystem (Fig. 3b, see also Francis et al. 2007). When such information is available—which is now true for a growing list of species and local populations, it should have tangible bearing on management advice and policy. To exemplify herein, we have summarized ‘Indices of vulnerability’ for the highlighted target species (see Table 1). Such traits must be considered carefully when designing harvest rules, where conservation of old-growth age structure should be a priority. Moreover, management needs to be implemented at relevant spatial scales, i.e., matching the geographical distribution of a population. We call for adaptive management systems including sub-stock monitoring and the use of management security fuses such as marine protected areas (MPAs). Adaptive management can be seen as a learning process with stock monitoring as an important part (Curtin and Prellezo 2010). Stakeholder involvement and local ownership is vital to increase society’s understanding of managing local stocks and ecosystems, and quantitative data from both monitoring and effectiveness of MPAs must be used.

By now, MPAs that impose strict limitations on fishing activities (e.g., no-take marine reserves) are proven as efficient management tools for species with limited home ranges and may therefore be effective also for northern temperate coastal fish stocks (Fenberg et al. 2012; Fernández-Chacón et al. 2015). MPAs also serve as reproductive refugia, effectively conserving spawning biomass and promoting recruitment if overfishing occurs despite other management efforts. Importantly, protection may confer trophic changes and ecosystem stability. Indeed, reversal of urchin dominance has been demonstrated in no-take MPAs with restored predator populations and size-structure in the Mediterranean (Guidetti and Sala 2007), Tasmania (Edgar et al. 2009) and California (Hamilton and Caselle 2015).

Long-term management success requires a holistic approach and whole-ecosystem perspective (Francis et al. 2007; Houde 2008; Curtin and Prellezo 2010). Barren and kelp forest states are stable, while shifts between them are discontinuous (Ling et al. 2015). Understanding of threshold and feedback factors preventing kelp recovery should therefore be part of management strategies (Filbee-Dexter and Scheibling 2014). Recovery of kelp forests on the southern border of the barren ground area since the 1990s (Norderhaug and Christie 2009) suggest that this is achievable when conditions are favorable. Further work to explore the large-scale grazing event described, and the hypothesis put forward herein, would benefit from an ecosystem-modeling approach in which these dynamics might be simulated. Shared understanding of lessons learned, and inclusion of novel management and research tools in effective adaptive management systems are necessary to increase our understanding, avoid future ecosystem collapses and to restore kelp forests and depleted coastal fish stocks.
